# Transforming social media text into predictive tools for depression through AI: A test-case study on the Beck Depression Inventory-II

**DOI:** 10.1371/journal.pdig.0000848

**Published:** 2025-06-05

**Authors:** Federico Ravenda, Antonio Preti, Michele Poletti, Antonietta Mira, Fabio Crestani, Andrea Raballo

**Affiliations:** 1 Faculty of Informatics, Università della Svizzera italiana (USI), Lugano, Switzerland; 2 Department of Neuroscience, University of Turin, Turin, Italy; 3 Department of Mental Health and Pathological Addiction, Child and Adolescent Neuropsychiatry Service, Azienda USL-IRCCS di Reggio Emilia, Reggio Emilia, Italy; 4 Faculty of Economics, Euler Institute, Università della Svizzera italiana, Lugano, Switzerland; 5 Department of Science and High Technology, Insubria University, Como, Italy; 6 Chair of Psychiatry, Faculty of Biomedical Sciences, Università della Svizzera italiana, Lugano, Switzerland; 7 Cantonal Sociopsychiatric Organisation, Public Health Division, Department of Health and Social Care, Repubblica e Cantone Ticino, Lugano, Switzerland; 8 Faculty of Biomedical Sciences, Euler Institute, Università della Svizzera italiana, Lugano, Switzerland; Regenstrief Institute Inc., UNITED STATES OF AMERICA

## Abstract

The characterization of mental states through assessment tools is a fundamental aspect in psychiatric and psychological clinical practice. In this context, standardized questionnaires based on Likert scales are often used for the assessment of emotions, attitudes, and perceptions. These tools enable clinicians and researchers to quantify subjective experiences, providing valuable data that elucidate the intricate nature of human emotions and beliefs. Despite their utility, administering and completing these questionnaires presents significant challenges. The process requires substantial time and resources from both clinicians and participants, which can create barriers to efficient data collection and analysis. Consequently, we aim to streamline this process without compromising the quality and reliability of the gathered data. This study was designed to develop a tool (aka EnsemBERT) that leveraging the power of Pre-trained Language Models (PLMs) could reliably predict the scores associated with each item of the Beck Depression Inventory (BDI-II) on the basis of users’ generated social media posts. The results confirm that such AI-based approach is feasible and that the specific tool, i.e. EnsemBERT, can accurately predict questionnaire scores at various levels of granularity, i.e. individual item scores as well as overall questionnaire scores.

## Author summary

Our study explores the potential of using social media activity to assess mental health, specifically by predicting scores on the Beck Depression Inventory-II (BDI-II) through the application of advanced natural language processing techniques. Traditionally, psychological assessments like the BDI-II require respondents to answer a series of questions, which can be time-consuming and resource-intensive. We aimed to simplify this process by developing a tool, EnsemBERT, that analyzes a person’s social media posts to predict their responses to the BDI-II. This approach leverages pre-trained language models to identify relevant social media content, enabling accurate predictions of depressive symptoms without the need for direct questionnaire administration. Our results indicate that this method is not only feasible but also highly accurate, suggesting it could be a valuable tool for real-time mental health monitoring. This work has significant implications for making mental health assessments more accessible and scalable and can pave the way for future studies on real-time mental health monitoring using digital footprints, potentially enabling earlier interventions and personalized care strategies.

## Introduction

Psychological questionnaires are crucial tools for understanding and diagnosing mental states within the field of mental health. They serve a role similar to that of biochemical tests in physical medicine. Just as biochemical tests help assess physical health by measuring substances in the body, psychological questionnaires help evaluate mental health by capturing and analyzing emotional, cognitive and psychobehavioral patterns. In particular, psychological assessment questionaries are meticulously designed to measure an extensive range of psychological constructs, including emotional states (e.g. anxiety, depression, and distress), personality traits (e.g. impulsivity, introversion, stress tolerance), social attitudes (e.g. interpersonal relationships, social support, and communication skills) or behavioral dimensions (e.g. sleep, eating habits, and physical activity).

Within the area of psychological assessment, an established and widely used method is the Likert scale [1], which measures the intensity of subjective experiences. Likert scales typically require respondents to rate their agreement with various statements on a gradient (e.g., from “strongly disagree” to “strongly agree”), capturing subtle variations in feelings and attitudes. This quantification facilitates data analysis [2, 3], enabling the identification of trends, correlations, and deviations within populations.

A well-known example of a likert-based, frequently used psychological questionnaire is the Beck Depression Inventory II (BDI-II) [4]. This self-report inventory assesses the severity of depressive symptoms, as presented in the Diagnostic and Statistical Manual of Mental Disorders – Fourth Edition [5] (DSM-IV), through 21 multiple-choice questions, each targeting specific symptoms such as *mood, pessimism, sense of failure, guilt, suicidal thoughts, agitation, social withdrawal, insomnia, and loss of libido*. Each item is scored on a scale from 0 to 3, with higher scores indicating more severe depression. The total score categorizes the intensity of the depressive state into minimal *(0-9)*, mild *(10-18)*, moderate *(19-29)*, and severe *(30-63)* depression. The BDI-II is extensively validated and widely used in both clinical and research settings to monitor treatment progress and screen for depression across diverse populations, providing a detailed profile of depressive symptoms.

The BDI-II includes a range of depressive symptoms that can be divided into four main dimensions [6], each relevant for assessing different aspects of psychological distress. Compared to its predecessor, the BDI-IA [7], BDI-II incorporates significant changes in the symptoms evaluated, and the phrasing of questions [8]. These changes align the BDI-II more closely with the modern diagnostic criteria of the DSM-IV.

Studies have shown that both self-disclosure and social support play crucial roles in enhancing perceived self-efficacy and improving the quality of life [9].

The rise of digital platforms and the increasing prevalence of social media have introduced new possibilities for psychological assessment. Computational methods for analyzing user-generated content on social media have shown significant promise in yielding insights into psychological states. Social media platforms offer unique opportunities to monitor psychological risks unobtrusively, as users often perceive these platforms as safe spaces to share their emotions and concerns [10, 11]. This results in the collection of authentic and unfiltered data about their mental state.

Research has highlighted that individuals with mental disorders frequently use social media to share personal experiences, seek information about mental health and treatment options, and provide or receive support from others facing similar challenges [10, 11].

Mental illness, however, is often associated with social stigma, leading individuals to be cautious about disclosing their condition [12, 13]. The anonymous nature of platforms like Reddit provides a unique opportunity to study such stigmatized illnesses. The dissociative anonymity afforded by Reddit can lead to a disinhibition effect, encouraging individuals to share more openly about their mental health experiences [14].

This has led to various initiatives aimed at health monitoring and surveillance on social media using natural language processing (NLP) techniques, such as eRisk [15].

The eRisk initiative focuses on the early detection of mental health conditions through the analysis of digital traces left by individuals online. eRisk organizes a series of challenges to develop and evaluate models capable of predicting psychological states based on social media activity. These challenges encompass a range of mental health issues, including depression, anorexia, and self-harm, providing a structured framework for the research community to advance methodologies in this emerging field. By leveraging these digital platforms, researchers can enhance their ability to monitor and address mental health conditions, offering timely interventions and support.

In this work, we build on the foundation of eRisk datasets by introducing a novel approach that uses social media posts to predict degrees of depression, using BDI-II scores as a psychometric descriptor. Our method integrates a retrieval technique to identify the most relevant social media posts for each inventory item w.r.t. each user, coupled with a neural architecture that leverages Pre-Trained Language Models (PLMs) to deliver highly accurate predictions. This approach not only addresses the complexities highlighted by the eRisk challenges but also provides a scalable solution to enhance the efficiency and accuracy of psychological assessments. By employing advanced NLP techniques, we aim to streamline the evaluation process, reducing the burden on both clinicians and patients, and potentially offering real-time monitoring and intervention for mental health conditions using a scalable and lightweight solution. Analysis and evaluation are guided by the transparent reporting of a multivariable prediction model for individual prognosis or diagnosis (TRIPOD) [16] guidelines.

### 0.1 Heuristic aims and focus

The primary aim of this work is to develop a comprehensive pipeline designed to retrieve and analyze social media posts for depression assessment and then testing its performative potential in terms of precise ascription of BDI-II scores. In other words for each user and each BDI-II item, we will test if our approach identifies the most relevant social media posts, which are then used as inputs for a neural model capable of predicting BDI-II scores. These predictions are made both at the level of individual items and the overall score, allowing us to accurately determine the severity of depressive states. We aim at demonstrating the feasibility of such approach and that the use of PLMs could boost accuracy in both retrieval and prediction phases. Further, we show how the choice of different PLMs impact the prediction performances.

### 0.2 The use of NLP approaches in the psychological domain: A quick overview

Recently, the use of NLP approaches in the psychological domain has seen a significant increase [17]. This surge in interest is largely due to the potential of NLP techniques to extract meaningful markers from spoken and written language, providing valuable insights into various mental health conditions [18–21]. For instance, studies have demonstrated that measures of language coherence can be powerful predictors of psychotic symptoms in individuals at high clinical risk [22]. More clearcut language production deficits are present in the first episode of psychosis [23]. Language disorganization, which can be assessed through the coherence and logical consistency of speech, is a key indicator of such symptoms. Techniques based on topic modeling [24] and matrix-factorization [25] have been employed to evaluate psychosis symptoms during patient interviews, analyzing the thematic structure and latent factors within the language to provide a nuanced understanding of the mental state of the patients [26].

The advent of deep learning, particularly approaches based on PLMs such as Transformers [27], has revolutionized the field. These models, pretrained on extensive corpora, capture and synthesize the semantics of text into dense and compact vector representations. These embeddings can be used in various contexts: in scenarios with limited data, they can serve as inputs for simpler models [28, 29], significantly boosting their performance, or for more specific applications, LLMs can be fine-tuned on domain-specific datasets, enhancing their ability to predict mental health disorders accurately [30].

Despite the extensive literature on detecting signs of mental health disorders, there are relatively few studies focused on modeling psychometric data related to depressive symptoms. One notable study use computational linguistic evaluation to predict self-reported symptoms of depression and anxiety through freely generated word responses, showing significant correlations between the generated responses and the diagnostic criteria of established scales such as PHQ-9 [31], GAD-7 [32], and PSWQ-8 [33]. Another study applied Latent Semantic Analysis (LSA) to evaluate responses to open-ended questions, demonstrating that LSA-based models can achieve validity and reliability comparable to, or even exceeding, traditional numerical rating scales. This approach was validated across nine different paradigms, highlighting its robustness and applicability.

Authors in [34] developed a predictive model based on Twitter behaviors, including social engagement, expressed emotions, and linguistic styles, achieving 70% accuracy in predicting depression before its clinical manifestation, while in [35], authors introduced DEPTWEET, a dataset of 40,191 tweets annotated for depression severity based on the PHQ-9 questionnaire. Their evaluation using various classification models showed that BERT-based models achieved the best performance, with an accuracy of 83.29% in symptom classification.

Also in [36], authors developed a PHQ-9-based approach for social media text analysis, creating classifiers capable of categorizing sentences according to the nine depression symptoms defined in the questionnaire. Their best model, based on BERT architecture, achieved 68.3% accuracy in detecting depression based solely on social media textual data.

Additional work [37] employed BERT embeddings alongside Ridge Regression and KNN to predict participants’ responses to Myers-Briggs Type Indicator (MBTI) questionnaire by analyzing their Facebook posts and survey questions. However, this approach presents limitations: creating user embeddings by averaging embeddings from various posts may result in inconsistent representations, as posts more relevant to answering a specific question receive the same weight as irrelevant ones. Finally in [38], authors implemented a model to predict the MBTI based on user-generated social media posts, but relies on a fixed number of posts per user, not taking into account for varying post numbers across users.

Compared to our scenario, the studies that use PHQ-9 and MBTI deal with binary data, presenting a simpler scenario compared to data referencing a Likert scale, as in our case.

Our approach distinguishes itself from previous work by combining advanced NLP techniques with a pipeline that leverages social media data to evaluate and predict depression levels according to BDI-II scores. Unlike the cited methodologies, our approach integrates a retrieval system to identify the most relevant posts for each questionnaire item and employs a novel neural architecture based on sophisticated language models to provide accurate predictions for both item scores and overall depression category.

The results of this study show that leveraging the power of PLMs, can help researchers and clinicians to achieve deeper insights into mental health conditions and develop more effective and timely interventions.

## 1 Materials and methods

In this section, we introduce the task problem and present the novel approaches used for inferring the scores of the BDI-II inventory.

### 1.1 Problem definition

We argue that the digital footprints left by users on social media platforms are sufficient to approximately identify the various dimensions of a standardized psychological test, such as the BDI-II.

Guided by this intuition, we propose a novel method that use the content of an individual’s Reddit posts to answer items in a psychological survey, thus enabling an assessment of their personality by correlating the textual data with survey questions.

**Fig 1:**
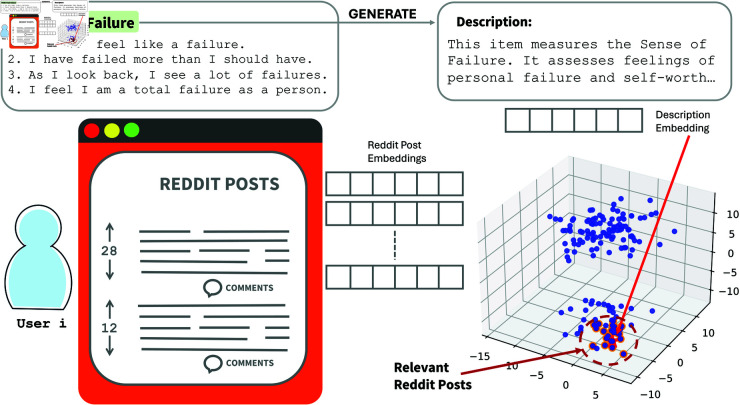
Given a user i in the bottom left, and a collection of Reddit posts, we generate a representation for each post. In the top left, an example item from the BDI-II is shown. Based on this item, a description is generated, to which an embedding is associated. Subsequently, the 20 posts most similar to the description, from a semantic similarity perspective, are retrieved.

Our goal is to implement a function that maps reddit posts and the context of survey items to predicted scores:


f(P,D,C)→S

where *P* denotes reddit Posts, *D* is the item Description, *C* represents item Choices, and *S* represents the item Score. Fig 1 provides an example of these three types of semantic information. To create this mapping, we use a neural approach (described in details in [arch_a]Sect 1.4), called EnsemBERT, which leverages the representational power of PLMs to enhance the accuracy of the scores associated with each item.

Given the variability in the number and relevance of posts per individual, we implement a retrieval strategy to find the *K* most relevant reddit posts to each survey item:


RP⊆P

In this context, *RP* are the Relevant Reddit Posts extracted from the initial pool of Reddit Posts.

Each survey item typically has multiple choices indicating different levels of intensity for a particular trait or symptom. For this reason, for each item, we retrieve the posts that are most similar to a specific description (see Fig 1 for a visual interpretation).

We implemented a workflow pipeline that begins with a variable number of posts for each user and a specific item for which we want to predict a score. The process involves retrieving the posts that are semantically most similar to the item, followed by using the EnsemBERT model to predict the score. The EnsemBERT architecture consists of two main sub-models and an ensemble model that combines their outputs to make final predictions. This hierarchical structure allows EnsemBERT to leverage both the contextual relevance of the posts and the alignment with survey choices, resulting in more accurate and interpretable predictions of the BDI-II scores.

### 1.2 Dataset

The datasets used in this study are from the *2019* and *2021* editions of eRisk, a series of tasks focused on the early detection of depression symptoms [39, 40]. These datasets contain Reddit posts from 100 users in total, each associated with a self-reported score for every item of the BDI-II questionnaire.

**Fig 2:**
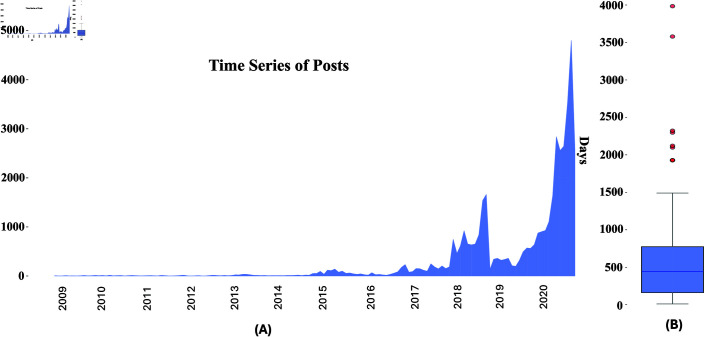
(A) show a time series visualization of the number of posts over time within the dataset. (B) show the distribution of posting timespan per user (number of days between their first and last post).

Table 1 and Fig 2 presents some characteristics of the dataset. Table 1 shows the frequency distribution of depression severity levels across users. We observe that users with Moderate and Severe depression states constitute a large portion of the total user base, while those with Minimal and Mild depression represent the minority. In Table 1 we see reported the absolute frequency distribution of scores within the dataset. There is a notable imbalance in the distribution: lower scores (0 and 1) account for 60% of all scores, while higher scores (2 and 3) represent 40%.

**Table 1:** Summary statistics of number of posts considered per user, number of users conditioned on depression intensity, and frequencies of the scores.

	Mean	Median	Std
Avg Posts/User	274	165	265
Depression Categories	**Level**	**Absolute Frequency**	
	Minimal	10	
	Mild	17	
	Moderate	31	
	Severe	42	
	**Total Users**	100	
Score Distribution	**Score**	**Relative Frequency (%)**	
	0	25	
	1	35	
	2	23	
	3	17	

Fig 2(A) presents the time series of user posts, while Fig 2(B) illustrates the distribution of posting timespan per user. In particular, Fig 2(A) shows that the majority of posts occurred in 2020, which can be attributed to the fact that most users were participants in the 2021 edition of eRisk.

Given the small number of users, we focus the analyses at the level of individual items, thereby increasing the number of samples in our dataset (each user will have 21 observations, corresponding to the number of items in the BDI-II). By focusing on this finer granularity, we not only increase the number of observations but we can also aggregate them to estimate the final score and give more reliable predictions.

Since the users’ posting history spans different time periods marked by COVID-19, rather than splitting users based on the two different editions, we employ a typical 80-20 holdout strategy.

Table 2 presents the descriptive statistics (mean and standard deviation) and item-total correlations for each questionnaire’s item for both the train and test datasets.

**Table 2:** Internal Reliability of the BDI-II by dataset splitting. Mean, Standard Deviation, Cronbach’s α, and Item-Total correlation for train (80) and test (20 users) are shown.

Item	Train			Test		
	Mean	Sth	Item-Total corr	Mean	Sth Dev	Item-Total corr
Sadness	1.17	0.80	0.52	0.95	1.05	0.89
Pessimism	1.41	1.04	0.56	1.20	1.11	0.69
Past failure	1.58	0.97	0.59	1.35	1.14	0.72
Loss of pleasure	1.40	0.84	0.66	1.05	0.69	0.62
Guilty feelings	1.13	1.06	0.58	1.40	1.14	0.45
Punishment feelings	1.00	1.15	0.60	1.00	1.12	0.75
Self-dislike	1.65	1.10	0.67	1.20	1.15	0.66
Self-criticalness	1.54	0.98	0.57	1.35	1.14	0.60
Suicidal thoughts or wishes	0.88	0.74	0.53	0.80	1.01	0.63
Crying	1.35	1.25	0.43	1.25	1.33	0.71
Agitation	1.10	0.82	0.42	1.10	1.07	0.63
Loss of interest	1.59	0.99	0.69	1.10	0.97	0.65
Indecisiveness	1.38	1.16	0.60	1.40	1.14	0.63
Worthlessness	1.38	1.13	0.74	1.35	1.23	0.82
Loss of energy	1.67	0.92	0.60	1.35	1.09	0.68
Changes in sleep pattern	1.46	1.03	0.51	1.45	0.89	0.79
Irritability	0.99	0.89	0.29	1.05	1.00	0.75
Changes in appetite	1.27	0.98	0.47	0.90	0.91	0.34
Concentration difficulty	1.51	0.90	0.67	1.55	0.94	0.58
Tiredness or fatigue	1.59	0.97	0.60	1.40	1.23	0.73
Loss of interest in sex	1.14	1.08	0.47	0.95	0.94	-0.05
**Cronbach’s α**	**0.92**			**0.94**		

The means and standard deviations offer information about the distribution of scores for each questionnaire item. The mean scores vary between the two datasets, with some differences.

The item-total correlations measure how well each item correlates with the total questionnaire score, excluding the item itself. Higher values indicate greater internal consistency of the item with the construct measured by the questionnaire. Some items show high correlations in both datasets, suggesting strong internal consistency. Item-total correlation is computed for each item by correlating its scores with the total questionnaire score derived from all other items (i.e., the total excluding the specific item). Higher values of item-total correlation indicate that the item aligns well with the overall construct being measured, suggesting that it is effectively capturing a dimension of the construct. For example, “*Loss of Interest*” has a correlation of 0.69 in the train and 0.65 in the test. Other items, such as “*Loss of interest in sex*”, show relatively low correlations (0.47 in the train and -0.05 in the test), suggesting they may not contribute as well to the overall construct measurement.

The results show that the questionnaire has good internal consistency, with high Cronbach’s alpha [41] values in both datasets (0.92 for the train and 0.94 for the test).

**Ethics statement.** Access to the data was obtained by following the standard procedures and the terms and conditions established by the creators of the eRisk dataset. It is also noted that the creators of the dataset obtained consent from the social media users during the data collection process.

Finally, during our research and analysis, we did not contact any social media users nor did we intervene in any way.

### 1.3 Retrieving relevant posts with a zero-shot dense retrieval approach

For each user, we have access to an extremely variable number of Reddit posts (we observe from Table 1 that, considering only posts with more than two words, the variability of the number of posts considered per user is quite large; in fact, the coefficient of variation, that expresses the relationship between the mean and the standard deviation, is 96.72%). Many of these posts are irrelevant to a particular item, and including them in the analysis as model input can introduce noise. Therefore, we employ a zero-shot Dense Retrieval approach, coco-dr [42], which has demonstrated state-of-the-art performance in several recent works [43–45]- to consider only the most relevant posts of an individual conditioned on a specific item.

To achieve this, we use a dense retrieval method (also shown in Fig 1): for each individual *i*, semantic embeddings are generated for both the items and the documents. For each item, we use its description to generate an embedding. Once the embeddings for the posts and items are obtained for each individual, we retrieve the 20 posts that are most similar to the item description based on the dot product:


$$\text{similarity}(E_{p},E_{item})=E_{p}\cdot E_{item}$$

Here, the dot product (represented by ·) is used to measure the similarity between the embedding of a post (*E*_*p*_) and the embedding of the item description (*E*_*item*_). We then select the top 20 posts with the highest similarity scores:


$$RP_{i}=\{p\in P_{i}\;|\;\text{top-20}(\text{similarity}(E_{p},E_{item}))\}$$

In this context, *RP*_*i*_ represents the set of Relevant Reddit Posts for individual *i*, selected based on their similarity to the item description embedding.

### 1.4 EnsemBERT architecture

**Fig 3:**
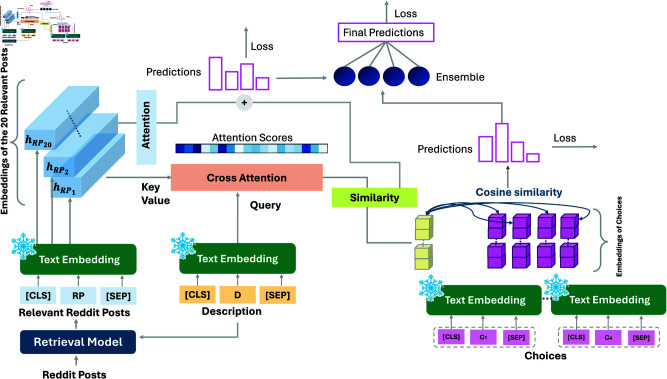
Overview of the EnsemBERT architecture.

**Fig 4:**
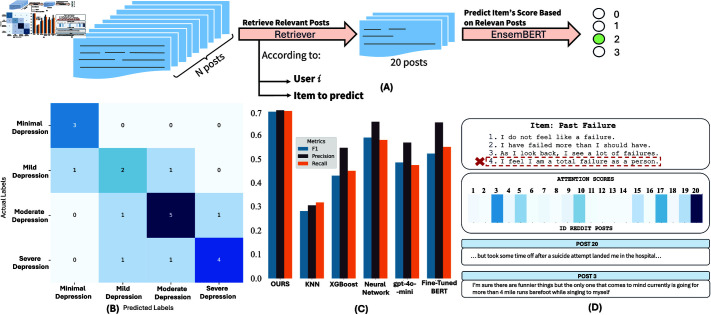
(A) Pipeline of work of our proposed approach. (B) Confusion matrix for the predictions on the test set regarding the depression intensity scores. (C) Bar plot of the F1, Precision, and Recall metrics for different models presented in Table 5. (D) An example of how Reddit posts are weighted for a specific item is illustrated. The model autonomously determines which posts are the most important and assigns higher weights to them for the final prediction.

Our architecture, illustrated in Fig 3, leverages three distinct sources of information: the descriptions of each item in the questionnaire, the four choices related to each item, and a variable number of Reddit posts for each user (our complete workflow process is illustrated in Fig 4(A)). The embeddings for each of these sources are computed using a pre-trained language model, with the best performances obtained using the SBERT model. To reduce the number of model parameters, the weights of the SBERT model are frozen, and the sentence representations are directly extracted.

Within our model, we can identify three different blocks, each associated with a different type of semantic information:

- **Reddit Posts**: For each individual, we have a variable number of Reddit posts. After identifying the most relevant posts for each individual according to each item, these posts are encoded using SBERT to generate embeddings, denoted as $RP\in {\mathbb{R}}^{k\times d}$, where *k* is the number of retrieved posts, set to 20, and *d* is the embedding dimension, which depends on the dimension of the language model used (dimensions are discussed in Sect 2).
- **Item Description**: Each questionnaire item has a description. In the BDI-II, the description consists of a few words characterizing the item (e.g., Sadness, Loss of Interest, etc.). To provide richer semantic content, these descriptions are expanded and enriched with the help of experts (two examples are shown in Table 3, all the items’ descriptions are available in the Github repository). The resulting embeddings have dimensions $𝐃\in {\mathbb{R}}^{d}$.
- **Item Choices**: Each item *j* (where j=1,2,…,21) has four choices, each encoded separately using SBERT, resulting in four embeddings ${𝐂}_{jl}\in {\mathbb{R}}^{d}$ for l=1,2,3,4.

**Table 3:** Examples of items and enriched descriptions.

Pessimism
This item measures Pessimism. It evaluates the individual’s outlook on the future and the presence of hopelessness, assessing whether the individual feels optimistic, discouraged, or hopeless about the future.
Punishment Feelings
This item measures the Sense of Punishment. It assesses feelings of being punished or expecting punishment, determining if the individual feels they might be punished or are being punished constantly.

Starting from the representations of the relevant Reddit posts, these are fed into a cross-attention layer along with the item description embedding (𝐃). This helps align the user’s Reddit activity with the specific description of the questionnaire item:


$$CA=\text{softmax}(𝐃\;{RP}^{T})RP$$

where **CA** represents the cross-attention output. To connect this combined information with the four choices related to the item, we calculate a similarity score between **CA** and each choice’s embedding. This score is obtained by computing the cosine similarity:


$$CS_{l}=\text{sim}(CA,C_{l})=\frac{CA\cdot C_{l}}{\left\|CA\|\|C_{l}\right\|}$$

These scores are concatenated and passed through a softmax layer to produce similarity values for each choice:


$$𝐒=\sigma ([{\text{CS}}_{1},{\text{CS}}_{2},{\text{CS}}_{3},{\text{CS}}_{4}])$$

where σ represents a softmax activation function. The output **S** represents a prediction of the score, adjusted via a categorical cross-entropy loss function.

The choice embeddings are weighted according to their similarity scores, and this weighted sum is then combined with the weighted mean of the relevant Reddit posts, where the weights are determined by the attention scores (denoted as α):


$$\left(\sum_{l=1}^{4}CS_{l}\cdot {𝐒}_{l}+\frac{1}{k}\sum_{i=1}^{k=20}\alpha_{i}\cdot {RP}_{i}\right)$$

The resulting combined output is fed into an output layer, which uses a softmax function to predict the final score.

Finally, the predictions from the two sub-models are fed into a ensemble model - a dense layer - that weighs these inputs and generates a final prediction. This hierarchical architecture enables EnsemBERT to seamlessly combine and use various sources of semantic information, leading to precise predictions of BDI-II scores.

## 2 Results

### 2.1 Experiments

Various language models were used to create representations passed as input to the EnsemBERT model. These include fastText [46], which generates non-contextual 300-dimensional embeddings; Bidirectional Encoder Representation Transformers (BERT) [47], which produces word-contextual 768-dimensional representations; and two Sentence-BERT (SBERT) - all-MiniLM-L6-v2 and all-mpnet-base-v2 - models [48, 49], which generate sentence-level representations of 384 and 768 dimensions respectively. In the first two cases, the embeddings generated from words are averaged to obtain a final sentence representation.

Five different metrics are used to evaluate the model’s performance: Accuracy, which is the percentage of correct predictions; F1 weighted score, which balances precision and recall to account for class imbalance; Mean Absolute Error (MAE), which measures the average absolute difference between predictions and actual values to measure average error magnitude; Mean Squared Error (MSE), which measures the average squared difference between predictions and actual values to capture sensitivity to larger errors; and Root Mean Squared Error (RMSE), which is the square root of the MSE, providing an indication of the model’s prediction error magnitude.

Scores are reported both at the item level (model’s goodness in predicting individual scores associated with each item, denoted with the subscript *item*) and the overall score (the sum of the scores associated with the different items for each individual, denoted with the subscript *overall*). Additionally, from the overall score, the intensity of the depressive state can be extracted and categorized into one of four levels (Minimal, Mild, Moderate, and Severe), with performance measures extracted accordingly (denoted with the subscript *category*). Specifically, to calculate the MSE_*category*_ score, Minimal depression is assigned a value of 0, Mild depression is assigned a value of 1, Moderate depression is assigned a value of 2, and Severe depression is assigned a value of 3. In particular, let *y*_*i*_ be the true overall BDI-II score for individual *i*. The depression severity category *c*_*i*_ is defined as: $c_{i}=\left\{ \begin{array}{cc} 0\text{ (Minimal)} & \text{if }0\leq y_{i}\leq 13\\ 1\text{ (Mild)} & \text{if }14\leq y_{i}\leq 19\\ 2\text{ (Moderate)} & \text{if }20\leq y_{i}\leq 28\\ 3\text{ (Severe)} & \text{if }y_{i}\geq 29 \end{array}\right.$

The MSE_*category*_ is then computed as: $\text{MSE}category=\frac{1}{N}\sum {i=1}^{N}(c_{i}-\hat{c_{i}}{)}^{2}$ where $\hat{c_{i}}$ is the predicted category for individual *i* and *N* is the total number of individuals.

Table 4 shows the results of the EnsemBERT approach with different language models used to generate embeddings. It can be observed that the SBERT models yield the best results overall. Specifically, for the item and questionnaire metrics, the best performance is achieved using all-mpnet-base-v2. In terms of the *overall* score, all-MiniLM-L6-v2 performs best in terms of RMSE_*overall*_, while fastText excels in terms of MAE_*overall*_. The worst results are obtained using the word-contextual representation model (bert-base-uncased).

**Table 4:** Results of the EnsemBERT model with different semantic language models. Scores are reported both at the item level and for the overall intensity score of the depressive state.

Language Model	MAE_*item*_	MSE_*item*_	MAE_*overall*_	RMSE_*overall*_	ACC_*category*_	F1_*category*_	MSE_*category*_
	Item Level		Questionnaire Score				
all-MiniLM-L6-v2	0.845	1.224	10.125	12.256	0.600	0.626	0.750
EnsemBERT	0.799	1.182	8.885	13.095	0.700	0.697	0.450
bert-base-uncased	0.912	1.456	11.234	15.467	0.400	0.393	1.300
fastText	0.817	1.184	8.750	13.489	0.600	0.585	1.150

Regarding the prediction of the categorization of depression intensity, the trivial model would predict all users as with “*Moderate Depression*”, achieving an accuracy of 35.0%, whereas our best model achieves an accuracy of 70.0%.

If we consider the MAE metric at the item level, a score of 0.799 per item - reached with the *all-mpnet-base-v2* SBERT model- means that, on average, the model’s predictions for each item in the BDI-II questionnaire are off by slightly more than half a point. Given that BDI-II item scores range from 0 to 3, this level of error is quite low, indicating that the model is making very accurate predictions for individual items. For the overall score, the best score is given by the *all-MiniLM-L6-v2* SBERT model. In this case a MAE of 8.885 means that the model’s predictions are, on average, about 8.8 points off when summing the scores across all BDI-II items, which range from 0 to 63. This is particularly reasonable considering that the BDI-II categories shift approximately every 10 points (e.g., minimal, mild, moderate, and depression). Therefore, even with this error, the model remains fairly accurate in assessing whether a person’s depression falls within the correct severity category, making it effective for distinguishing between different levels of depression severity.

To highlight the goodness of our approach, in Table 5 we present a comparison of our proposed EnsemBERT method with different benchmark models, including both shallow machine learning methods - KNN [50], known for its simplicity and interpretability, XGBoost [51], well known for its excellent predictive performance, and a feed-forward neural network - and more advanced models - a fine-tuned version of BERT, where the most relevant posts are concatenated and separated by the special token [*SEP*] as input, and an LLM approach based on gpt-4o-mini model used as a zero-shot classifier.

**Table 5:** Results of the EnsemBERT and benchmark models. In this case, all models leverage all-mpnet-base-v2 as the semantic model to extract embeddings used as input for the models. Scores are reported both at the item level and for the overall intensity score of the depressive state.

Model	MAE_*item*_	MSE_*item*_	MAE_*overall*_	RMSE_*overall*_	ACC_*category*_	F1_*category*_	MSE_*category*_
	Item Level		Questionnaire Score				
EnsemBERT	0.799	1.182	8.885	13.095	0.700	0.697	0.450
KNN	1.110	2.052	13.400	18.216	0.300	0.282	1.600
XGBoost	0.850	1.486	10.029	13.086	0.450	0.430	0.850
Neural Network	0.831	1.302	9.032	14.593	0.550	0.589	0.900
gpt-4o-mini	0.821	1.326	10.567	14.065	0.450	0.485	1.050
Fine-Tuned BERT	0.879	1.319	11.567	14.859	0.550	0.522	1.150

With respect to shallow learners, the mean of the embeddings of the relevant posts is used as input to the model. Since the best results for EnsemBERT were obtained using all-mpnet-base-v2, we use embeddings generated from this model for the benchmark models to ensure a fair comparison. In all cases, except for the MAE_*overall*_, EnsemBERT outperforms the benchmark models when using the same embeddings. For gpt-4o-mini, we used the retrieved documents for each user relative to each item and incorporated them into the prompt, instructing the LLM to predict the score of the specific item based on the retrieved posts (RAG-based approach). With the exception of RMSE_*overall*_, EnsemBERT outperforms all the benchmark models for all the considered metrics.

In Fig 4(B), we observe the confusion matrix of the predictions on the test set. It shows that, for the category Severe Depression, the highest severity of the condition is correctly predicted in 4 out of 6 cases. Moderate intensity is correctly predicted in 5 out of 7 cases, the condition Mild is misclassified twice, and the minimal condition is correctly classified in all the cases.

Additionally, Fig 4(C) shows a comparison between EnsemBERT and the different implemented models on the final depression state categorization in terms of Precision, Recall, and F1 score, in order to take into account class imbalance. We observe that our model performs better than others in correctly identifying severity across different classes.

### 2.2 Interpretation of results

Since EnsemBERT employs attention mechanisms in its predictions, we can examine how the model weights different parts of the input data. The attention mechanism assigns varying importance to different Reddit posts in relation to each psychological survey item.

Through the Cross-Attention mechanism, the model learns to weigh the relevance of each post against the description of specific psychological items. Following established approaches for model interpretability [52, 53], we leverage attention scores to quantify how much each post contributes to the prediction of individual items.

Fig 4(D) illustrates this process. For example, when examining the ‘Past Failure’ item where the user reported the highest severity level, we can identify which posts most influenced the model’s assessment. Posts 20 and 3 received notably higher attention weights, indicating their stronger contribution to this prediction.

The model’s output is designed to align with the BDI-II clinical framework, mapping predictions directly to established risk categories (minimal, mild, moderate, and severe). While this design choice enhances the clinical utility of our results by enabling interpretation through standard BDI-II scoring guidelines, we acknowledge that such outcome interpretability differs from full model interpretability and explainability.

## 3 Discussion

The results of our study using EnsemBERT highlight several insights and implications. By leveraging advanced NLP techniques and dense retrieval methods, EnsemBERT demonstrates a robust ability to predict depression levels with high accuracy, as evidenced by its performance across various metrics at a different granularity.

One of the critical advantages of EnsemBERT is that it is a scalable and lightweight method capable of leveraging all the semantic information present in a questionnaire and social media posts to improve predictions. EnsemBERT uses attention mechanisms and similarity functions, allowing the model to focus on the most relevant parts of the input data and relate them to the elements of the questionnaire (description and choices).

Attention mechanisms operate by assigning varying degrees of importance to different sections of the text, enabling the model to prioritize significant content over less relevant information. In the context of our study, this means that EnsemBERT can effectively discern which Reddit posts are most pertinent to predict the score of a given item in the BDI-II questionnaire. This approach not only enhances the accuracy of the predictions but also provides a mechanism for interpreting the model’s decisions.

Furthermore, our study reveals that different language models used for generating embeddings significantly impact the model’s performance. The SBERT models, particularly all-mpnet-base-v2, provided the best results for item and questionnaire metrics.

Looking at the results of all the models used, both the approach proposed and the benchmarks, we observe that our hypothesis— i.e., that it is possible to predict the scores of items in a standardized psychological questionnaire like the BDI-II using the semantic information from Reddit posts—is empirically validated. By focusing on a subset of Reddit posts relevant to a specific survey’s question and leveraging the semantic characteristics of both the questionnaire and the Reddit posts, it is possible to establish a link between the Reddit content and the item scores.

The architecture we implemented, compared with benchmark models, further validates the strengths of EnsemBERT. The consistent outperformance of EnsemBERT across most metrics highlights the efficacy of our approach.

Overall, our study highlights the potential of integrating advanced NLP techniques and PLMs into psychological assessments, offering a scalable and efficient solution for real-time monitoring and intervention.

### 3.1 Strengths and limitations

In this work, we have demonstrated that it is possible to infer responses to a psychological questionnaire with high accuracy using the historical social media posts of users. Our method is effective both at the level of individual item scores and in categorizing each user into one of the depressive categories.

The proposed model, EnsemBERT, is highly customizable (various language models can be explored for creating semantic embeddings), lightweight, and scalable. Moreover, it provides interpretable results, making it a versatile tool for psychological assessments.

This model is not limited to the BDI-II questionnaire, but can also be easily extended to other types of questionnaires, offering a broad range of applications beyond the scope of this study. The major limitation of this work rely on the relatively small sample size of participants. This could limit the generalizability of the findings. Although predicting individual item scores increases the data sample size by leveraging multiple observations per user, the overall number of unique participants remains limited. Nonetheless, the proof-of-concept value of the current results provides the rationale for future studies with larger and more diverse participant pools to further validate and extend the findings.

Additionally, while our current approach uses attention weights to provide some insight into the model’s behavior, this offers only limited interpretability. The attention mechanisms, while useful for understanding which posts influence predictions more heavily, do not provide comprehensive explanations for the model’s decisions.

Addressing these limitations through further research and development will be essential in enhancing the model’s accuracy, robustness, and applicability across diverse settings and populations. Additionally, to enhance model explainability, possible future works should incorporate more sophisticated post-hoc explanation methods (such as LIME or SHAP), integrate domain expertise in the evaluation of model explanations, and potentially explore ante-hoc development approaches that prioritize interpretable features.
